# Mild and Short-Term Caloric Restriction Prevents Obesity-Induced Cardiomyopathy in Young Zucker Rats without Changing in Metabolites and Fatty Acids Cardiac Profile

**DOI:** 10.3389/fphys.2017.00042

**Published:** 2017-02-01

**Authors:** Gema Ruiz-Hurtado, Concha F. García-Prieto, Helena Pulido-Olmo, Juan P. Velasco-Martín, Palmira Villa-Valverde, María E. Fernández-Valle, Lisardo Boscá, María Fernández-Velasco, Javier Regadera, Beatriz Somoza, María S. Fernández-Alfonso

**Affiliations:** ^1^Unidad de Hipertensión, Instituto de Investigación Imas12, Hospital Universitario 12 de OctubreMadrid, Spain; ^2^Facultad de Farmacia, Instituto Pluridisciplinar, Universidad Complutense de MadridMadrid, Spain; ^3^Departamento de Ciencias Farmacéuticas y de la Salud, Facultad de Farmacia, Universidad CEU-San PabloMadrid, Spain; ^4^Departamento de Anatomía, Histología y Neurociencia, Facultad de Medicina. Universidad Autónoma de MadridMadrid, Spain; ^5^CAI de RMN y RSE, Facultad de Químicas, Universidad ComplutenseMadrid, Spain; ^6^Departamento de Metabolismo y Señalización Celular, Instituto de Investigaciones Biomédicas Alberto Sols (CSIC-UAM)Madrid, Spain; ^7^Departamento de Metabolismo y Señalización Celular, Instituto de Investigación Hospital Universitario La Paz (IdiPAZ)Madrid, Spain

**Keywords:** caloric restriction, hypertrophy, hypertension, nuclear magnetic resonance, metabolite profile, obesity-induced cardiomyopathy

## Abstract

Caloric restriction (CR) ameliorates cardiac dysfunction associated with obesity. However, most of the studies have been performed under severe CR (30–65% caloric intake decrease) for several months or even years in aged animals. Here, we investigated whether mild (20% food intake reduction) and short-term (2-weeks) CR prevented the obese cardiomyopathy phenotype and improved the metabolic profile of young (14 weeks of age) genetically obese Zucker *fa/fa* rats. Heart weight (HW) and HW/tibia length ratio was significantly lower in *fa/fa* rats after 2 weeks of CR than in counterparts fed *ad libitum*. Invasive pressure measurements showed that systolic blood pressure, maximal rate of positive left ventricle (LV) pressure, LV systolic pressure and LV end-diastolic pressure were all significantly higher in obese *fa/fa* rats than in *lean* counterparts, which were prevented by CR. Magnetic resonance imaging revealed that the increase in LV end-systolic volume, stroke volume and LV wall thickness observed in *fa/fa* rats was significantly lower in animals on CR diet. Histological analysis also revealed that CR blocked the significant increase in cardiomyocyte diameter in obese *fa/fa* rats. High resolution magic angle spinning magnetic resonance spectroscopy analysis of the LV revealed a global decrease in metabolites such as taurine, creatine and phosphocreatine, glutamate, glutamine and glutathione, in obese *fa/fa* rats, whereas lactate concentration was increased. By contrast, fatty acid concentrations in LV tissue were significantly elevated in obese *fa/fa* rats. CR failed to restore the LV metabolomic profile of obese *fa/fa* rats. In conclusion, mild and short-term CR prevented an obesity-induced cardiomyopathy phenotype in young obese *fa/fa* rats independently of the cardiac metabolic profile.

## Introduction

Obesity is a strong independent risk factor for cardiovascular (CV) disease and is associated with an increased CV mortality (Adams et al., 2006; Adabag et al., 2015). The prevalence of obesity in childhood and adolescence is reaching epidemic proportions. Moreover, it has been recently shown that increased body mass index in late adolescence, even within a clinically accepted normal range, is strongly associated with higher CV mortality in young adulthood or midlife (Twig et al., 2016). Unless this clinical situation is addressed at an early stage in life, it is likely that adverse CV outcomes will occur as a consequence of cardiac damage resulting from overweight and obesity.

Despite the well-established link between obesity and CV risk, the pathogenesis of obesity-induced cardiomyopathy is not fully understood. Several factors as deleterious metabolic and structural remodeling contribute significantly to the subsequent functional cardiac damage. In this sense, in the obese-induced cardiomyopathy the heart undergoes a metabolic remodeling in response to the high energy demand characterized by a reduction of the energy reserve compounds as phosphocreatine and by a lipid overload, both conditions impairing cardiac efficiency (Kolwicz et al., 2013). These metabolic changes observed in hearts with obese-induce cardiomyopathy occur early and often precede later functional changes as ventricular dysfunction (Peterson et al., 2004). Thus, the most common structural and physiological changes of the heart in obesity include cardiac hypertrophy and diastolic dysfunction (Alpert, 2001; Rider et al., 2014), and both are associated with all-cause mortality (Levy et al., 1988; Bhatia et al., 2006).

Several strategies are available to protect the heart from obesity-induced cardiomyopathy, with caloric restriction (CR) as one of the most common, cost-effective non-pharmacological and non-surgical interventions used to reduce body weight (BW) and CV risk. CR is defined as a state in which energy intake is reduced below normal *ad libitum* (AL) intake without malnutrition (García-Prieto and Fernández-Alfonso, 2016). CR increases longevity and improves the outcome of obesity-associated diseases, including CV disease (Fontana et al., 2010). Clinical and experimental studies have shown that CR ameliorates cardiac dysfunction associated with obesity, although the majority of these studies were carried out under one or more of the following conditions: (1) high to severe CR regimen (30–65% reduction of food intake; Zheng et al., 2009; Niemann et al., 2010; Shinmura et al., 2011; Takatsu et al., 2013; Melo et al., 2016); (2) long-term CR (several months) (Meyer et al., 2006; Hammer et al., 2008; Zheng et al., 2009; Niemann et al., 2010; Melo et al., 2016); and (3) adult or elderly individuals (Meyer et al., 2006; Hammer et al., 2008; Niemann et al., 2010; Shinmura et al., 2011). The potential benefits of a milder shorter-term CR regimen on hearts with obese-induced cardiomyopathy in a younger population are much less known. Testing a mild CR regimen and in young animals would help to elucidate the relative contribution that changes in the metabolic, structural and functional remodeling have on obese cardiomyopathy in at early stage of development. Thus, the purpose of the present study was to investigate the impact of mild (20% food intake reduction) and short-term (2 weeks) CR on the cardiac phenotype in young genetically obese Zucker rats through the parallel analysis of cardiac function, cardiomyocyte structure and metabolic profile.

## Materials and methods

### Animals and caloric restriction protocol

**Figure 1:**
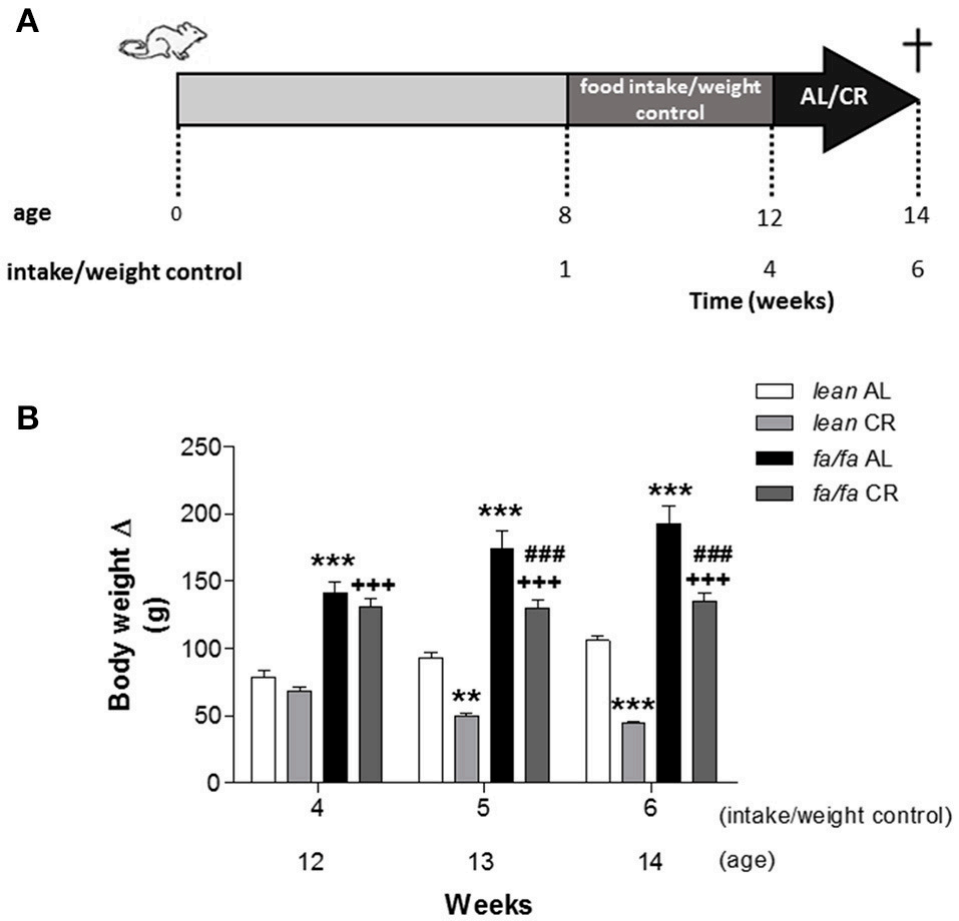
**Scheme of experimental protocol (A)** and body weight gain **(B)** of *lean* and *fa/fa* rats with and without caloric restriction. Half of the animals of each experimental group were fed with 80% of their daily food intake (CR) and the other half were maintained on AL diet. Data are mean±SEM (*n* = 8 animals per group). ^**^P < 0.01 and ^***^P < 0.001 vs. *lean* AL group; ^+++^*P* < 0.001 vs. *lean* CR group; ^###^*P* < 0.001 vs. *fa/fa* AL group.

Eight-week old male obese Zucker rats (*fa/fa)* and non-obese control (*lean*) were housed under specific controlled dark-light cycles (12/12h, lights on at 8:00 a.m.) and temperature (22°C), with standard chow and water *ad libitum* (AL). To control BW and food intake, animals were housed individually and daily monitored for 4 weeks. Then, animals were randomly divided into two groups and assigned either to an AL or to a CR diet (80% of AL) for two additional weeks. A scheme of the protocol is shown in Figure 1A. Adjustment of 20% CR was done individually based on the previous food intake values. Rats were weighed before sacrifice. All animal procedures were conducted in accordance with the recommendations of the Spanish Animal Care and Use Committee according to the guidelines for ethical care of experimental animals of the European Union (2010/63/EU). The study was approved by the Ethical Committee of Universidad Complutense and Comunidad de Madrid (reference: PROEX 413/15).

### Hemodynamic parameters

Rats were anesthetized with ketamine (Imalgene 1000, Merial; 80 mg/kg i.p.) and xylazine (Rompun 2%, Bayer; 8 mg/kg i.p.), and a 0.58/0.97 mm inner- and outer-diameter, respectively, catheter was inserted in the left ventricle (LV) through the right carotid artery. The catheter was connected to a data acquisition system (PowerLab 4/30, ADInstruments, UK) and signals were digitally stored for analysis using the LabChart 7.0 Pro software. Systolic (SBP) and diastolic (DBP) blood pressure, LV systolic pressure (LVSP), LV end-diastolic pressure (LVEDP), the first derivative of LV pressure rise over time (dP/dt_max_), and the first derivative of LV pressure decline over time (dP/dt_min_) were analyzed. After hemodynamic measurements, animals were sacrificed and hearts were removed for study. Hearts were weighed and heart weight (HW)/tibia length ratio was used as an index of cardiac hypertrophy.

### Magnetic resonance imaging acquisition and analysis

Magnetic resonance imaging (MRI) was performed with a Biospec BMT 47/40 spectrometer (Bruker, Ettlingen, Germany) operating at 4.7-Teslas, equipped with a 12-cm gradient system. Rats were anesthetized with an isoflurane and oxygen mixture (3% in oxygen at 1.5 L/min for induction and 1.0–1.5% at 1.0 L/min during experiments). Body temperature was monitored and maintained at 36°C. Heart rate (HR) and respiration were monitored and used to trigger image acquisition with 1025 SAM monitoring and gating system (SA Instruments, Inc., USA). Several gradient echo images with different orientations were acquired to localize the short axis planes. Images were cardiac and respiratory triggered and up to 7 slices were acquired in a cardiac cycle. Repetition time (RT) was variable depending on the animal's heart and respiration rate. Other imaging parameters were as follows: echo time (ET) = 2.7 ms; flip angle (θ) = 80°; field of view (FOV) = 6 × 6 cm^2^; slice thickness = 2.0 mm; matrix size = 128 × 128; number of averaged images = 2. Once the short axis was set, a multislice white blood CINE sequence was used to image the rat's entire heart. For these experiments, a cardiac and respiratory-triggered FLASH sequence was used. Ten images per cardiac cycle were acquired to completely cover the cardiac cycle. Other imaging parameters were as follows: ET = 2 ms; θ = 80°; FOV = 5.12 × 5.12 cm^2^; slice thickness = 1.5 mm; matrix size = 128 × 128; number of averaged images = 8. A total of 8–10 slices were acquired to cover the whole heart. The data were transferred to a PC for analysis, which was carried out with ImageJ 1.49v (NIH, USA). For each slice of the CINE sequence, the LV was segmented in the images corresponding to diastole and systole. The LV volume for each slice was summed to obtain the total left ventricular (LV) volume at the end of diastole and systole (EDV and ESV, respectively). The heart rate for each rat was calculated as the mean value of the heart rate during the entire CINE experiment. Ejection fraction (EF), stroke volume (SV), and cardiac output (CO) were obtained from these data applying these formula: EF = [EDV-ESV/LVEDV]^*^100; SV = EDV-ESV; CO = SV ^*^ HR

### Histological analysis

Hearts were fixed in 4% buffered formaldehyde solution for 48 h. Hearts were sectioned transversally including both ventricles. Cardiac sections were embedded in paraffin. Histological sections were obtained at 5 μm and stained with periodic acid solution and Schiff's reagent. Cardiomyocytes of the left ventricular wall were photographed in three high power fields (× 40 objective) for each animal. The minor diameter of cardiomyocytes was measured. We only measured those cardiomyocytes with a recognizable nucleus. Diameter lower than 13 μm were rejected since they were considered as a dichotomy branch of cardiomyocyte; and diameters higher than 30 μm were rejected because borders between cardiomyocyte were not well delimited. The cardiomyocyte diameter was obtained using the morphometric ImagenJ software.

### Real-time PCR for mRNA collagen type I analysis

Total RNA was extracted from frozen LV tissue with TRIzol reagent (Invitrogen) and 1 μg was reverse transcribed using the Transcriptor First Strand cDNA Synthesis Kit for RT-PCR (Roche). Real-time PCR was performed on a MyiQ Real-Time PCR System (Bio-Rad) using Taqman Gene Expression Assays (Applied Biosystem). PCR thermocycling parameters were 50°C for 2 min, followed by 40 cycles of 95°C for 15 s and 60°C for 1 min. Expression of type I collagen was measured using the Quanti SYBR Green RT protocol (Roche). Melting curve data were collected to check the PCR specificity. Each cDNA was amplified in triplicate and the corresponding sample without reverse transcriptase (no-RT sample) was included as a negative control. Gene expression levels were normalized to that of 36B4 mRNA. Replicates were then averaged, and fold induction was determined using a ^ΔΔ^Ct-based fold-change calculation. The primers used were type I collagen (forward primer 5′-AAT GGC ACG GCT GTG TGC GA-3′, reverse primer 5′- AGC ACT CGC CCT CCC GTC TT-3′) and 36B4 (forward primer 5′-AGA TGC AGC AGA TCC GCA T-3′), (reverse primer 5′-GTT CTT GCC CAT CAG CAC C-3′).

### Metabolites and fatty acids quantified by ^1^H HR-MAS NMR

High-resolution magic angle spinning (^1^H HR-MAS) NMR spectroscopy was performed at 500.13 MHz using a Bruker AMX500 spectrometer 11.7 T (Bruker Rheinstetten, Germany) on frozen LV samples placed within a 50 μl zirconium oxide rotor with cylindrical insert and spun at 4000 Hz spinning rate. Standard solvent suppressed spectra were acquired into 16 k data points, averaged over 256 acquisitions, total acquisition ~14 min, using a standard Bruker sequence (noesypr1d) with a relaxation delay of 2 s and a mixing time of 150 ms. A spectral with of 8333.33 Hz was used. All spectra were processed using TOPSPIN software, version 1.3 (Bruker). Prior to Fourier transformation, the FIDs were multiplied by an exponential weight function corresponding to a line broadening of 0.3 Hz. Spectra were phased, baseline-corrected and referenced to the sodium (3-trimethylsilyl)-2,2,3,3-tetradeuteriopropionate singlet at δ 0ppm. Several ^1^H, ^1^H 2D experiments were performed to carry out the component assignments. Gradient selected COSY90 was acquired under the following conditions: water presaturation during relaxation delay, spectral with of 8333 Hz in both dimensions, 2 k data points in f_2_ and 384 increments in f_1_. An unshifted sinusoidal window function was applied in both dimensions and zero filling in f_1_ dimension. ^1^H, ^1^H TOCSY was registered in TPPI phase-sensitive mode, with water presaturation during 2 s relaxation delay, a spectral with of 8333 Hz in both dimensions, a 70 ms mixing time, 2 k data points in f_2_ and 384 increments in f_1_. Zero filling in f_1_ and unshifted squared sinusoidal window function in both dimensions were applied before Fourier transformation.

### Statistical analysis

Multivariate statistical algorithms were used to classify ^1^H HR-MAS NMR spectra of LV samples and identify distinct metabolic profiles for the different tissues. For pattern recognition analysis, ^1^H NMR spectra were data reduced using the software program AMIX (Analysis of MIXtures version 3.6.8, Bruker) by subdivision into integral regions of 0.04 ppm between δ 0.4 and 9 ppm (excluding the water region from 5.2 to 4.7 ppm). Individual integral regions were normalized to the total sum of integral region following exclusion of the water resonance. Principal component analysis (PCA) was applied to the data. Loadings plots from the PCA were used to identify the peaks mainly responsible for the significant differences. Peaks were therefore associated with specific metabolites based upon existing literature. Results were expressed as mean ± SEM. All comparisons were carried out using Student's *t*-test or one-way analysis of variance with a Neuman-Keuls *post hoc* test to compare between groups. Statistical significance was accepted at *P* < 0.05. Analysis was performed with GraphPad Prism 5.0 software.

## Results

### Caloric restriction and macroscopic parameters

Before CR, both *lean* AL and *fa/fa* AL animals gained weight, but BW increase was significantly higher in *fa/fa* rats compared to *lean* ones at week 12 (Figure 1B), related to cumulative food intake was also significantly higher in *fa/fa* AL rats compared to *lean* AL ones (Supplemental Figure 1; *P* < 0.001). During CR, BW remained stable in *fa/fa* CR rats, whereas it was significantly reduced in *lean* CR animals (*P* < 0.001). At the end of the study (14 week-old animals, data summarize in Supplemental Table 1), BW was significantly higher in *fa/fa* AL rats compared to lean AL ones, and significantly decreased in both groups after CR. However, tibia length (TL) was similar between all groups, suggesting that CR reduces adipose tissue amount but does not affect rat growth.

### Assessment of cardiac function

**Figure 2:**
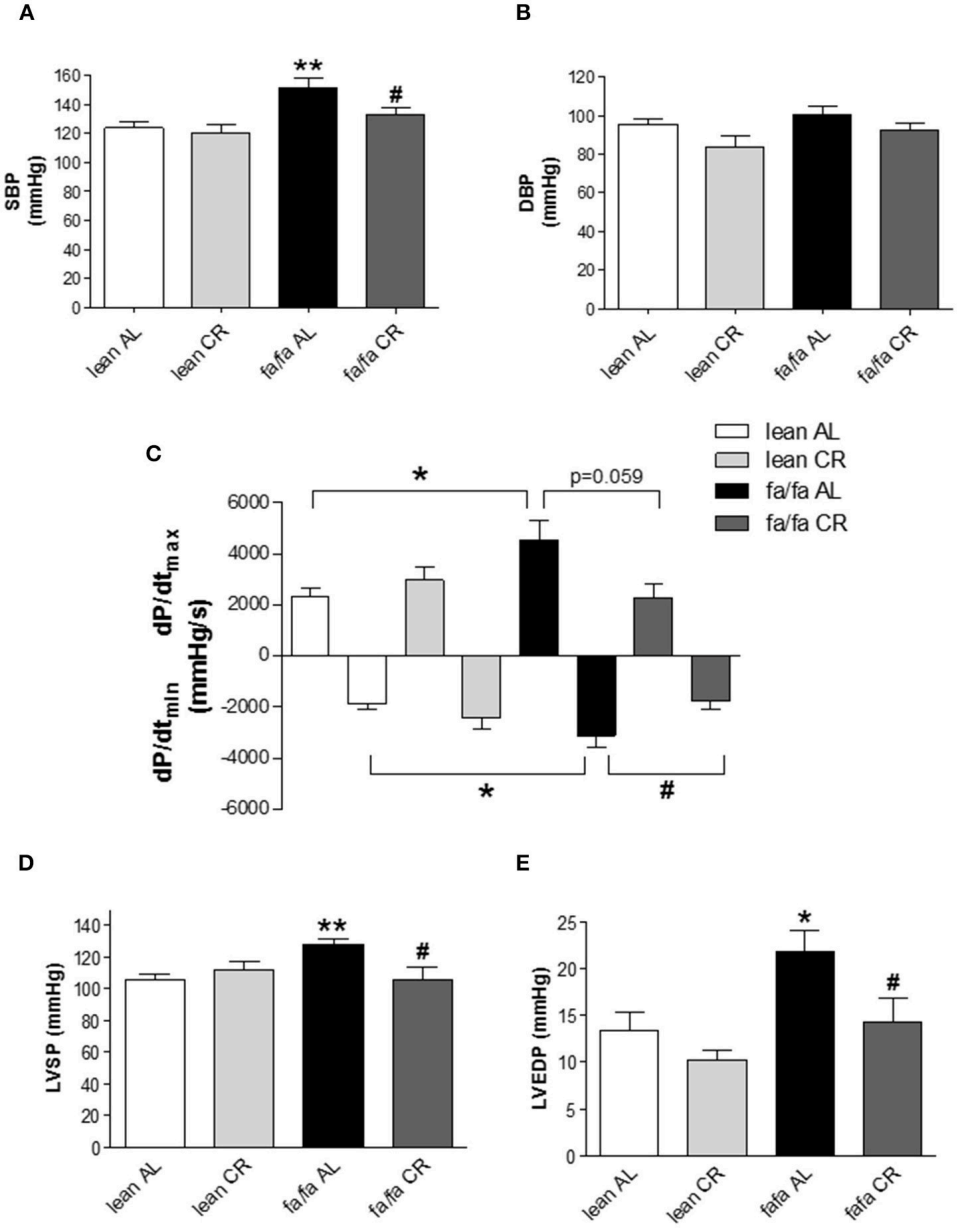
**Left ventricular pressure characteristics of *lean* and obese *fa/fa* rats with and without caloric restriction. (A)** Systolic blood pressure (SBP) and **(B)** diastolic blood pressure (DBP) in *lean* and *fa/fa* rats on AL or CR diet. **(C)** Maximal rate of pressure increase during systole (*dP/dt*_max_) and maximal rate of pressure decay during diastole (*dP/dt*_min_) in *lean* and *fa/fa* rats on AL and CR diet. **(D)** Peak left ventricular systolic pressure (LVSP) and **(E)** Left ventricular end-diastolic pressure (LVEDP) in *lean* and *fa/fa* rats on AL or CR diet. Data are mean±SEM (*n* = 7–12 animals per group). ^*^P < 0.05, ^**^P < 0.01 vs. *lean* AL rats; ^#^*P* < 0.05 vs. *fa/fa* AL rats.

Invasive pressure measurements showed that SBP was significantly higher in *fa/fa* AL than in *lean* AL rats (152.1 vs. 124.2 mmHg, *P* < 0.01; Figure 2A), and this increase was prevented in *fa/fa* CR rats (133.4 mmHg; *P* < 0.05 compared with *fa/fa* AL rats). No differences were found for DBP among groups (Figure 2B). Both *dP/dt*_max_ and *dP/dt*_min_ were significantly higher in *fa/fa* AL than in *lean* AL rats (*P* < 0.05; Figure 2C). CR normalized the magnitude of *dP/dt*_max_ and *dP/dt*_min_ in *fa/fa* CR rats to the level seen in the *lean* CR rats, being the values of both parameters lower in *fa/fa* CR rats (*P* = 0.059 and *P* < 0.05, respectively; Figure 2C). LVSP (Figure 2D) and LVEDP (Figure 2E) were significantly higher in *fa/fa* AL than in *lean* AL rats (*P* < 0.01 and *P* < 0.05, respectively), and both were normalized in *fa/fa* CR rats (*P* < 0.05).

**Figure 3:**
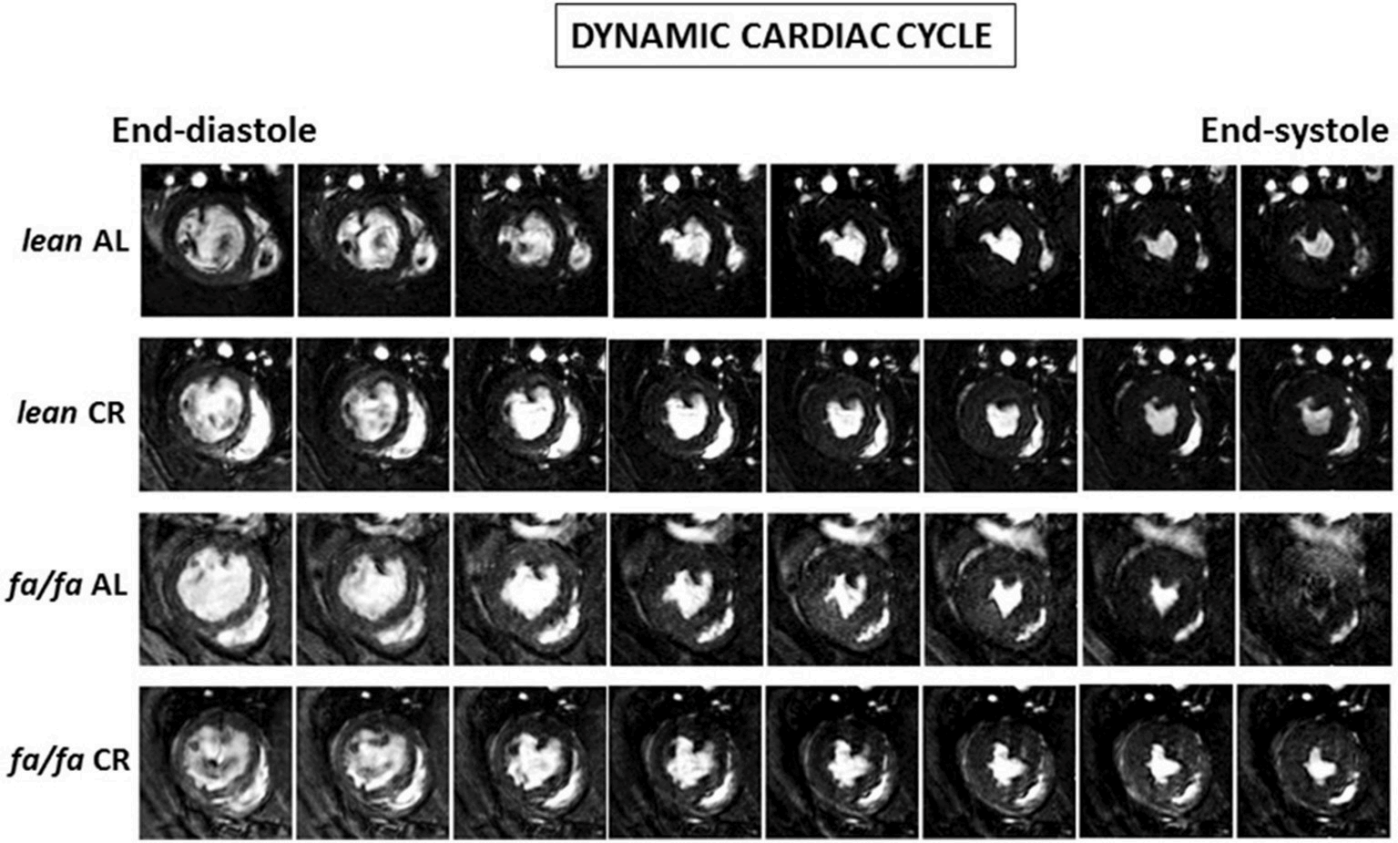
**Cardiac cycle MRI images**. Representative transverse mid-papillary MR sections along the cardiac cycle obtained from *lean* and obese *fa/fa* rats with and without short and moderate caloric restriction.

The cardiac MRI images of midventricular slides throughout cardiac cycle are shown in Figure 3. No differences were found in heart rate (HR) among groups during MRI analysis (Table 1). LVEDV but not LVESV, was higher in *fa/fa* AL than in *lean* AL rats (*P* < 0.05; Table 1), and as a consequence, SV and CO were also significantly higher in *fa/fa* AL rats (*P* < 0.05; Table 1). CR normalized the magnitude of LVEDV in *fa/fa* CR rats to the level seen in the *lean* CR rats (*P* < 0.05), and SV significantly decreased in *fa/fa* CR rats (*P* < 0.05). No differences were found in EF between *lean* and *fa/fa* groups of rats independently of food intake applied (Table 1).

**Table 1:** **Cardiac functional parameters determined by MRI**.

**Parameters**	***lean* AL**	***lean* CR**	***fa/fa* AL**	***fa/fa* CR**
HR (bpm)	275.8±15.5	307.7±17.3	284.7±18.4	293.2±15.1
LVEDV (μL)	374.5±36.4	387.9±12.7	494.3±23.8^*^	399±33.2^#^
LVESV (μL)	127.3±8.6	116.0±6.3	113.5±3.9	108.4±3.1
EF (%)	68.8±2.7	70.1±1.5	76.9±1.2	72.4±2.0
SV (μL)	256.9±26.0	271.9±11.5	380.8±22.7^*^	290.7±31.8^#^
CO (mL/s)	1.25±0.16	1.40±0.12	1.77±0.07^*^	1.55±0.23

*Data are mean ± SEM (N = 4–5 for each group). HR, heart rate; LV, left ventricular; LVEDV, LV end-diastolic volume; LVESD, LV end-systolic volume; EF, ejection fraction; SV, stroke volume; CO, cardiac output*.

* P < 0.05 vs. lean AL;

# *P < 0.05 vs. fa/fa AL*.

### Cardiac structure and cardiomyocyte size

**Figure 4:**
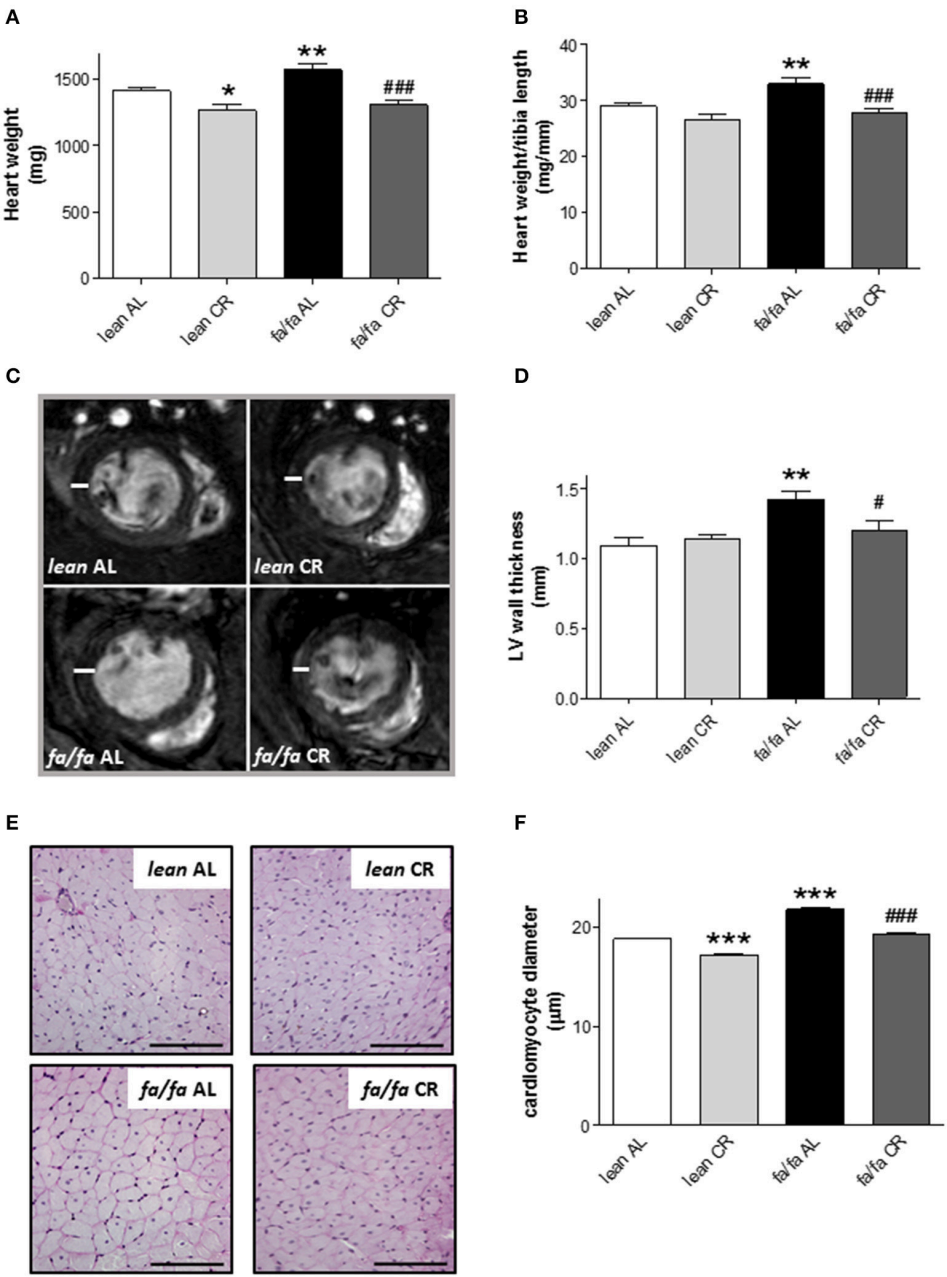
**Heart dimensions and cardiomyocyte diameter of *lean* and obese *fa/fa* rats with and without short and moderate caloric restriction. (A,B)** Heart weight (HW) **(A)** and HW to tibia length (TL) ratio (HW/TL) **(B)** of each group of animals. **(C)** Representative transverse mid-papillary MR sections during end diastole from *lean* and obese *fa/fa* rats with and without short and moderate caloric restriction. **(D)** Quantitative analysis of LV free wall thickness of each group of animals. Data are mean±SEM (*n* = 5 animals per group). ^*^P < 0.05 and ^**^P < 0.01 vs. *lean* AL rats; ^#^*P* < 0.05, ^###^*P* < 0.001 vs. *fa/fa* AL rats. **(E)** Representative images of cardiomyocyte cross-surfaces obtained from *lean* and *fa/fa* rats with and without short and moderate caloric restriction (bar: 100 μm). Micrographs of cardiomyocyte sections were stained with PAS reagent. **(F)** Quantitative analysis of cardiomyocyte diameter measured in μm of each group of animals. Data are mean±SEM (*n* = 5 animals per group). ^***^ *P* < 0.005 vs. *lean* AL rats; ^###^*P* < 0.005 vs. *fa/fa* AL rats.

HW (Figure 4A) and HW/tibia length ratio (Figure 4B) were significantly higher in *fa/fa* AL than in *lean* AL rats (*P* < 0.01). After CR, HW was significantly lower in both *fa/fa* CR and *lean* CR groups, although HW/tibia length ratio was significantly lower only in *fa/fa* CR compared with *lean* CR rats (*P* < 0.001; Figures 4A,B).

LV wall thickness was also greater in *fa/fa* AL than in *lean* AL rats (*P* < 0.01; Figures 4C,D), and this parameter was significantly lower in *fa/fa* CR rats (*P* < 0.05).

Histological examination of heart tissue (Figure 4E) illustrated the extent of cardiac hypertrophy in *fa/fa* AL rats, which was characterized by an increase in the diameter of cardiomyocytes in *fa/fa* AL rats relative to *lean* AL counterparts (*P* < 0.005; Figure 4F). Cardiomyocyte size was lower both in *fa/fa* CR and *lean* CR rats (*P* < 0.005; Figure 4F). To assess whether cardiac hypertrophy was associated with myocardial fibrosis, collagen deposition and collagen I gene levels were analyzed in *lean* AL and *fa/fa* AL rats. There was no evidence of pathological fibrosis in Masson's trichrome-stained sections of LV (data not shown). Moreover, no differences were detected in the relative expression of collagen type I mRNA between *lean* AL and *fa/fa* AL rats as measured by quantitative PCR (19.53 ± 0.49 vs. 21.35 ± 0.92 arbitrary units, respectively, *P* = 0.117).

### Myocardial metabolites and fatty acids profile

**Figure 5:**
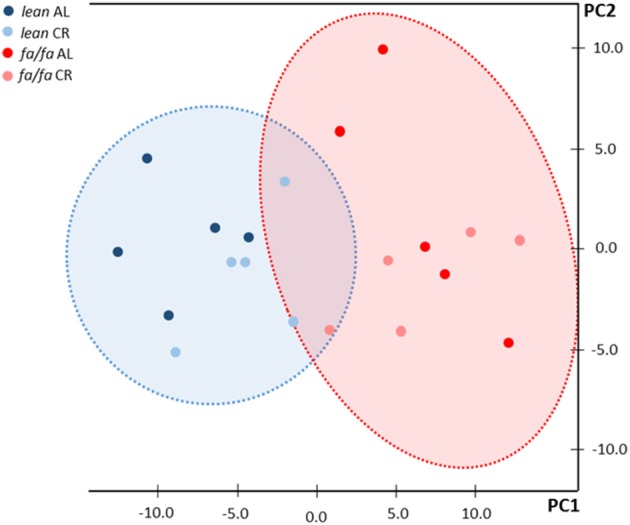
**Principal component analysis (PCA) of NMR data**. Blue circle corresponds to *lean* rats and red circle corresponds to *fa/fa* rats. Dark blue dots: *lean* AL rats; light blue dots: *lean* CR rats; dark red dots: *fa/fa* AL rats; light red dots: *fa/fa* CR rats.

^1^H HRMAS NMR spectra showed characteristic signals from many low molecular weight metabolites, including taurine, creatine plus phosphocreatine, glutamate, glutamine, glutathione, alanine and lactate, in LV extracts of each group (Table 2), as well as fatty acids including n-3 acyl chains, unsaturated and poly-unsaturated fatty acids (Table 3). ^1^H HRMAS NMR analysis of the cardiac metabolites profile revealed a significant decrease in the concentration of taurine (*P* < 0.01), creatine plus phosphocreatine (*P* < 0.001), glutamate (*P* < 0.01), glutamine (*P* < 0.001), and glutathione (*P* < 0.01) in *fa/fa* AL rats relative to *lean* AL counterparts. By contrast, lactate concentration was significantly higher in *fa/fa* AL rats than in *lean* AL counterparts (*P* < 0.001; Table 2). With respect to unsaturated fatty acids, in particular n-3 acyl chains, unsaturated and poly-unsaturated fatty acids, were all significantly higher in *fa/fa* rats than in *lean* counterparts (*P* < 0.01 and *P* < 0.001 Table 3). All these changes observed in both metabolites and fatty acids cardiac contect in *fa/fa* AL were unchanged in *fa/fa* CR rats. Therefore, mild and short-term CR does not change the cardiac metabolic profile of genetically obese *fa/fa* rats. In fact, the nonsupervised PCA showed a clear clustering of two groups, one for all *lean* animals (AL and CR; see dark and light blue dots into the blue circle of Figure 5), and another for all *fa/fa* animals, irrespective of whether that they were on AL or CR diets (Figure 5; see dark and light red dots into the red circle).

**Table 2:** **Metabolites identified by ^1^H HRMAS NMR from the Principal Component Analysis (PCA)**.

	***lean* AL**	***lean* CR**	***fa/fa* AL**	***fa/fa* CR**
Taurine (3.42)	44.3±1.42	45.9±2.92	31.84±1.62^**^	32.45±2.96^++^
Creatine^+^Phosphocreatine (3.03)	64.3±1.67	61.7±4.03	42.2±1.28^***^	43.1±3.14^+++^
Glutamate (2.33)	3.6±0.22	3.1±0.18	2.5±0.16^**^	2.3±0.11^+^
Glutamine (3.78)	14.8±0.44	13.9±0.51	9.6±0.56^***^	10.2±0.79^+++^
Glutamine/Glutamate	4.2±0.32	4.5±0.30	3.9±0.14	4.4±0.34
Glutathione (4.58)	2.1±0.10	1.9±0.21	1.4±0.07^**^	1.2±0.05^++^
Alanine (1.47)	4.5±0.17	4.4±0.25	3.8±0.24	4.0±0.38
Lactate (1.33)	61.5±2.64	66.0±2.54	82.2±4.22^***^	86.0±2.94^++^

*Data are mean ± SEM (n = 5 for each group)*.

** *P < 0.01*,

*** P < 0.001 vs. lean AL;

+ *P < 0.05*,

++ *P < 0.01*,

+++ *P < 0.001 vs. lean CR. Number between brackets correspond to the middle point of the bucket*.

**Table 3:** **Fatty acids identified by ^1^H HRMAS NMR from the Principal Component Analysis (PCA)**.

	***lean* AL**	***lean* CR**	***fa/fa* AL**	***fa/fa* CR**
n-3 acyl chains	36.5 ± 1.68	43.5 ± 4.86	60.7 ± 2.31^**^	63.2 ± 5.82^++^
Unsaturated fatty acids	2.5 ± 0.33	2.7 ± 0.37	5.7 ± 0.38^***^	5.2 ± 0.59^+++^
Poly-unsaturated fatty acids	1.7 ± 0.05	1.7 ± 0.17	2.3 ± 0.11^**^	2.1 ± 0.03^+^

*Data are mean ± SEM (n = 5 for each group)*.

** *P < 0.01*,

*** P < 0.001 vs. lean AL;

+ *P < 0.05*,

++ *P < 0.01*,

+++ *P < 0.001 vs. lean CR*.

## Discussion

The major findings of the present study can be summarized as follows: (1) short-term (2 weeks) and mild (20%) CR is sufficient to prevent cardiac dysfunction in young obese *fa/fa* rats; and (2) cardiac improvement associated with mild CR occurs even in the presence of a cardiometabolic profile characteristic of obesity.

Several factors including metabolic, structural and functional remodeling are involved in the development of obese-induced cardiomyopathy. Hemodynamic analysis and cardiac MRI were used to assess *in vivo* cardiac function. Whereas no changes in HR were detected between groups, LV cavity, LV wall thickness, LVEDV, SV and CO were all significantly higher in young obese *fa/fa* AL rats than in *lean* AL counterparts. However, this occurred despite of unchanged LVESV and EF, indicating that compensatory hypertrophy occurred in young obese *fa/fa* AL rats in response to increased overload by higher SBP. Moreover, both *dP/dt*_max_ and *dP/dt*_min_ were increased in *fa/fa* AL rats, which indicates an enhancement of LV contractility and distensibility as a compensatory mechanism of cardiac function. Collectively, these changes in cardiac function are characteristic of early stage obese cardiomyopathy. Our results are in concordance with the majority of studies on obese populations in which no effect on global measures of systolic function, such as LVESV or EF are observed (Powell et al., 2006; Rider et al., 2014). Prevention at this early stage is very important in terms of avoiding advanced obese cardiomyopathy characterized by LV systolic dysfunction. In the present study, we show that compensatory cardiac hypertrophy and related functional changes in *fa/fa* rats under AL diet can be prevented by CR diet, likely due to a lower hemodynamic overload normalizing SBP, LVSP and LVEDP values, and consequently parameters of cardiac function including LVEDV and SV. Therefore, our results point out that the prevention of an increase in systolic BP in fa/fa CR rats was the most probably candidate for avoiding the subsequent cardiac alterations. In addition to compensatory functional changes in *fa/fa* rats, structural changes also occurred in the whole heart and at the level of cardiomyocytes. Thus, HW/tibia length ratio, LV wall thickness and cardiomyocyte diameter were all significantly increased in *fa/fa* AL than in *lean* AL rats, and all were prevented by mild short-term CR. Clearly, the lack of deleterious fibrosis in *fa/fa* rats probably favored the rapid normalization of hemodynamic parameters and cardiac function.

With respect to metabolites profile, it has been proposed in recent years that impairment in myocardial phosphate energetics, such as creatine and phosphocreatine depletion, can occur in cardiac hypertrophy associated with obesity (Rider et al., 2012, 2014). A decline in the total creatine pool together with elevated fatty acid levels induce an important increase in mitochondrial uncoupling, which can lead to diastolic dysfunction (Diamant et al., 2003; Faller et al., 2013). This scenario is evident in young obese *fa/fa* rats. We observed a general decrease in the cardiac concentration of taurine, creatine and phosphocreatine, glutamate, glutamine, and glutathione linked to the obesity background of the animals. It has been previously described that a general drop in the concentration of these metabolites might be explained by the increase in the extracellular mass due to fibrotic tissue accumulation and excessive collagen deposition (Roncalli et al., 2007). However, collagen type I expression was at controls levels in hearts of *fa/fa* AL rats and no areas of interstitial fibrosis were detected. Supporting these results, *fa/fa* AL rats had a similar glutamine to glutamate ratio to *lean* AL rats. A decrease in this ratio would be expected in cardiac fibrosis because transforming growth factor beta, a key regulator of collagen production, upregulates phosphate-dependent glutaminase (Roncalli et al., 2007). Therefore, our results support the notion that hearts of obese young *fa/fa* AL rats are no more fibrotic than their *lean* AL counterparts, and the alterations in metabolite profile probably occur only at the level of the cardiomyocyte. The exception to the general drop in metabolites was for lactate, whose concentration was significantly higher in *fa/fa* rats than in *lean* rats. This increase in cardiac lactate together with a significant decrease in glutamate implies a shift to a glycolytic phenotype in *fa/fa* rats. Moreover, the accumulation of lactate derived from pyruvate oxidation has been related to an elevated energy demand as for example during increased muscle exercise (Goodwin and Taegtmeyer, 2000), a situation closely associated with the compensatory cardiac hypertrophy observed in young obese *fa/fa* rats. On the other hand, fatty acids are also a predominant substrate used in the adult myocardium (Kolwicz et al., 2013). In our study n-3 fatty acyl changes, unsaturated and poly-unsaturated, significantly accumulated in obese young *fa/fa* rats. Therefore, both conditions, i.e. a decrease in the creatine pool and an accumulation of fatty acids, might be directly responsible for cardiac dysfunction, especially at the diastolic level observed in *fa/fa* rats. Remarkably, we observed that the alteration in metabolites and fatty acids were maintained in *fa/fa* rats despite normalization of cardiac structure and function by mild short-term CR. Another possibility to explain these results might be the fa/fa rats phenotype. This genetic obesity model develops insulin resistance, which could be also present during CR, explaining the presence of similar cardiac metabolic profile observed in fa/fa AL and CR rats. In addition, results from non-supervised PCA indicated that the main change in the metabolic profile was not attributable to differences related to cardiac hypertrophy, hypertension or cardiac diastolic dysfunction, but rather to obesity background. All these results indicate that the recovery of myocardial energetic balance is not necessary to re-establish an adequate cardiac structure and function, suggesting that the beneficial CV effects of a short and mild CR are independent from metabolic remodeling, at least in an early stage of obesity. Therefore, the cellular hypertrophic response at the early stage of obese cardiomyopathy in young *fa/fa* rats is probably secondary to increased wall stress forced by cavity dilatation and increased filling pressures, and not to metabolic remodeling.

It is important to consider that, in contrast to lean rats CR avoided the weight gain in fa/fa rats. This maintenance of BW during the 2 weeks of CR was associated with only a moderate loss of BW in fa/fa CR relative to fa/fa AL rats at the end of the study. However, this absence of weight gain in fa/fa CR rats probably favored that these rats did not develop hypertension and the subsequent cardiac alterations associated with a compensated hypertrophy. Therefore, mild CR provides clinically valuable cardioprotective effects in terms of structure and function, and can prevent obese cardiomyopathy without the need for extreme weight loss. In contrast to our approach, the majority of published studies using experimental models where cardiac benefits are linked to CR were carried out under more severe CR regimens (30–65%), during long-term (several months), and/or on aging-animals (Dolinsky et al., 2010; Niemann et al., 2010; Shinmura et al., 2011; Finckenberg et al., 2012; Takatsu et al., 2013; Kobara et al., 2015; Melo et al., 2016). Along the same line, human obese cardiomyopathy in adulthood complicated by the presence of hypertension is associated with alterations in cardiac structure and function (Meyer et al., 2006; Hammer et al., 2008). In this setting, long-term CR during several months (Hammer et al., 2008) or even years (Meyer et al., 2006) as well as after bariatric surgery (Ikonomidis et al., 2007) has been demonstrated to improve myocardial function especially at the diastolic level. However, CR regimes for prolonged periods and/or with an appreciable reduction in food intake are difficult to implement and sustain in the obese population.

Finally, it is important to note that alterations in cardiac function and morphology present in obese adults are also apparent in the younger obese population. Thus, obesity, hypertension and concentric cardiac hypertrophy are independent predictors of diastolic dysfunction in obese children and adolescents (Dhuper et al., 2011). In this sense, the present study together with recent results from our group that demonstrate a restoration in impaired endothelial dysfunction in obese Zucker rats (García-Prieto et al., 2015), offer some insight into the effects that mild and short-term CR have in terms of CV prevention in obesity at an early stage. Our results support data from other studies that affirm that the positive benefits of CR are greater when sooner CR starts (Han and Ren, 2010; Melo et al., 2016) and, as demonstrated in the present study, CV benefits can occur without changes in the cardiac metabolic profile. One limitation of this study is not having cardiac pre-CR values in fa/fa animals in order to demonstrate whether the mild and short CR applied here was able to prevent or revert cardiac damage. Nonetheless, it is very likely that fa/fa AL rats did not have an established obesity-induced cardiomyopathy just before starting CR at 12 weeks of age, supporting the assumption that the mild and short-term CR applied here would be able to prevent the development of cardiac damage in these animal.

In summary, our results indicate that mild and short-term CR protects hearts from obesity-related cardiomyopathy even when the cardiometabolic profile remains altered. Therefore, early dietary intervention with a moderate CR of easy compliance when obese cardiomyopathy is first detected might reverse key cardiac alterations linked to obesity.

## Author contributions

Conceived and designed the experiments: GR-H, BS and MSF-A; performed experiments: GR-H, CG-P, HP-O, J-VM, PV-V, MEF-V, MF-V; analyzed the data: GR-H, JR, MF-V, CG-P, HP-O, PV-V, BS, MSF-A; contributed reagents/materials/analysis tools: GR-H, LB, BS, JR, MSF-A; wrote the paper: GR-H and MSF-A.

### Conflict of interest statement

The authors declare that the research was conducted in the absence of any commercial or financial relationships that could be construed as a potential conflict of interest.
