# Supplementary material for: Mild and Short-Term Caloric Restriction Prevents Obesity-Induced Cardiomyopathy in Young Zucker Rats without Changing in Metabolites and Fatty Acids Cardiac Profile
Source: Front Physiol. 2017 Feb 1;8:42. doi: 10.3389/fphys.2017.00042 (PMC5285365; doi:10.3389/fphys.2017.00042)
Supplement: Supplementary file 1 [file Table1.docx]

**SUPPLEMENTAL MATERIAL**

**Supplemental Figure 1. Time course of cumulative food intake.** During 4 weeks of intake/weight control in *lean* and *fa/fa* animals on *ad libitum* (AL) diet. ****P* < 0.001 vs. *lean* animals.

**Supplemental Table 1. Body weight (BW) and tibia length (TL)**

| Parameters | *lean* AL | *lean* CR | *fa/fa* AL | *fa/fa* CR |
| --- | --- | --- | --- | --- |
| BW (g) | 382.2 ± 8.14 | 319.9 ± 5.09^***^ | 524.7 ± 9.76*** | 491.2 ± 10.74^##, +++^ |
| TL (mm) | 48.6 ± 0.05 | 47.9 ± 0.06 | 47.7 ± 0.05 | 46.9 ± 0.07 |

Data are mean ± SEM (n = 12–19 for each group). ****P* < 0.001 *vs*. *lean* AL; ^##^*P* < 0.01 *vs*. *fa/fa* AL; ^+++^*P*<0.001 *vs*. *lean* CR.
